# Cortical Plasticity After Surgical Tendon Transfer in Tetraplegics

**DOI:** 10.3389/fnhum.2018.00234

**Published:** 2018-06-18

**Authors:** Knut Wester, Leiv M. Hove, Roger Barndon, Alexander R. Craven, Kenneth Hugdahl

**Affiliations:** ^1^Department of Clinical Medicine K1, University of Bergen, Bergen, Norway; ^2^Department of Neurosurgery, Haukeland University Hospital, Bergen, Norway; ^3^Department of Orthopaedic Surgery, Haukeland University Hospital, Bergen, Norway; ^4^Department of Radiology, Haukeland University Hospital, Bergen, Norway; ^5^Department of Biological and Medical Psychology, University of Bergen, Bergen, Norway; ^6^Division of Psychiatry, Haukeland University Hospital, Bergen, Norway

**Keywords:** cortical plasticity, functional MRI, motor cortex, spinal cord transection, tetraplegia

## Abstract

**Background:** Developmental cortical plasticity with reorganization of cerebral cortex, has been known to occur in young and adult animals after permanent, restricted elimination of afferent (visual or somatosensory) input. In animals, cortical representation of unaffected muscles or sensory areas has been shown to invade the neighboring cortex when this is deprived of its normal sensory input or motor functions. Some studies indicate that similar cortical plasticity may take place in adult humans.

**Methods**: In patients with a high cervical spinal cord injury leaving the patient without any movements of the fingers, we performed fMRI studies of the cortical representation of an elbow flexor muscle before and after a surgical procedure that changed its function to a thumb flexor, thus providing the patient with a useful grip.

**Results**: Preoperatively, the elbow flexion movement was elicited from a cortical area corresponding with the “elbow area” in healthy individuals. Despite the fact that an elbow flexor was used for the post-operative key-grip, this movement in the tetraplegic patients was elicited from a similar brain region as in healthy controls (the “hand area”). This supports our hypothesis that control of that muscle shifts from a brain region typically associated with elbow movement, to one typically associated with wrist movements.

**Conclusion**: The findings presented here show with fMRI that the human cortex is capable of reorganizing itself spatially after a relatively acute change in the periphery.

## Introduction

Developmental cortical plasticity, such as reorganization of the sensory cortex, has for the last four decades been known to occur in young, developing animals after permanent, restricted elimination of afferent (visual or somatosensory) input (Van der Loos and Woolsey, 1973; Woolsey and Wann, 1976; Hubel et al., 1977; Gilbert, 1998). The subsequent demonstration of early critical periods (“time windows”) in life for this plasticity generated the widespread assumption that sensory cortex plasticity didn’t occur in grown-up individuals. This belief, however, turned out to be wrong; even in adult animals the cortical representation of unaffected muscles or sensory areas tend to invade the neighboring cortex, if this is deprived of its normal sensory input or motor functions (Merzenich et al., 1983; Robertson and Irvine, 1989; Kaas et al., 1990; McCandlish et al., 1996). The neural mechanisms behind such sensory and motor cortical reorganization have been discussed in several reviews (Kaas et al., 1983; Buonomano and Merzenich, 1998; Gilbert, 1998; Irvine, 2010; Gilbert and Li, 2012). Cortical reorganization has also been shown to occur in humans after peripheral nerve injury. In one of the first studies in human, Mogilner et al. (1993) used magnetoencephalography (MEG) and found cortical reorganization over a 3–9 mm distance in the somatosensory cortex after surgery. Similarly, Langer et al. (2012) found a decrease in cortical thickness in the somatosensory area and reduced white matter integrity after limb immobilization using structural MRI and diffusion tensor imaging (DTI) (see also Gaetz et al., 2017 who measured changes in cortical somatotopic maps after limb amputation). Using functional magnetic resonance imaging (fMRI), Hlustík et al. (2004) found increase in primary motor and sensory areas after a 3-weeks motor-skills training program.

More specifically, Lotze et al. (1999, 2006) found in adult humans that the elbow area, but not the hand or mouth area, moved into cortical areas that usually represent parts of the body below the transection in patients with complete, thoracic spinal cord transections. Thus, their studies indicate that the cortical representation of the elbow moved into the cortical areas that were deprived of their sensory input by the spinal cord transection. Using a transcranial magnetic stimulation (TMS) technique, Irlbacher et al. (2002) could in a similar fashion demonstrate that the cortical motor representation of an elbow flexor (m. biceps brachii) had moved into the “vacant” hand area in humans with an amputation at the forearm level.

None of the human studies referred to above have dealt with cortical remodeling after acute changes in sensory input or motor functions. They have all studied such changes in chronic patients, long after acquisition of the neurological deficit. In this article, we present a model that allows us to study the effect on cortical organization after surgically induced changes in motor function of the arm and hand, in addition to the empirical results.

## Materials and Methods

### The Model

In brief, we have studied the cortical representation of an elbow flexor muscle (m. brachioradialis) before and after a surgical procedure that changes its function to a thumb flexor in patients with a high cervical spinal cord injury that has left the patient without any movements of the fingers. This surgical procedure then provides the patients with a useful grip.

### Subjects

Data herein were obtained pre- and post-operatively between April 2010 and June 2013 from four tetraplegic patients. Patients were all male, mean age 29 (range: 15–41) years at the time of surgery and the preoperative test, which was performed 1 day before scheduled surgery; they will hereafter by identified as P1–P4. Three of the patients (P1–P3) had a complete traumatic spinal cord transection at the level of the fifth cervical vertebra (C 5), leaving them without any movements in the hand or fingers. The last patient (P4) had an incomplete spinal cord transection, leaving him with a severely weakened hand grip.

P1 and P2 were operated on the right side, with the aim to provide a key-grip function. P3 had earlier been operated on the right side to provide a key-grip function, and therefore underwent a similar operation on the left side. Thus, pre- and post-operative data collected from the left arm actions by P3 represent an equivalent condition to data collected from P1 and P2 on right arm actions, while data collected from right arm action on P3 reflect a longer-term post-operative state for that side. P4 was operated in a slightly different manner, to reinforce an existing, however weak, hand grip on the right side.

Additionally, control data was obtained from three healthy, non-operated subjects: all male, mean age 34 (*SD*: 8) years. These will be referred to as C1–C3.

### Surgery

Surgical reconstruction is an established method to restore grip and grasp function in patients who have lost these functions after traumatic spinal cord injury and tetraplegia. Musculus brachioradialis (BR) is the most important muscle to reconstruct active thumb flexion as part of a pinch grip or as a part of more complex hand reconstructions.

The brachioradial muscle is an elbow flexor with proximal origin at the distal humerus, which distally inserts into bone at the radial styloid, thus not extending beyond the wrist. Its motor function is limited to elbow flexion, a function shared with the biceps and brachialis muscles. When reconstructing active grip function, the BR-tendon is released distally and attached to the tendon of the paralytic long thumb flexor, thus performing a brachioradialis-to-flexor pollicis longus (BR-FPL) tendon transfer (Lamb and Landry, 1971; Waters et al., 1985). This reconstruction changes the function of the BR-muscle from an elbow flexor to mainly a thumb flexor, and thereby gives the patient an active “key grip” (pinch grip) function as the thumb now can be pressed against the radial side of the index finger. After the transposition of the BR tendon, this muscle no longer takes part in elbow flexion; this movement is now performed solely by the biceps and brachialis muscles.

### Post-operative Training

Post-operatively, the patients must practice the new use of the muscle. To achieve the key grip, they will initially flex the elbow, thereby also moving the thumb in a key grip. With time, they gradually experience that elbow flexion no longer is necessary, and the key grip is performed disconnected from elbow flexion. This gradual shift of motor control usually takes place within months.

### MR Imaging

For several practical reasons, the patients could not be taken back for the post-operative fMRI session as soon they had learned the key grip. The post-operative fMRI sessions for patients 1–4 took place 12, 27, 3, and 9 months after surgery, respectively.

Patients were watched very closely during the post-operative “key grip” fMRI sessions. If they obtained the key grip by “cheating,” i.e., still flexing the elbow, the session was discontinued and the patient was taken back for a new post-operative fMRI when they were able to perform the key grip without elbow flexion. This was the case for P2, who was first attempted tested 6 months after surgery, but needed more training before he mastered the task and was therefore taken back 27 months after for the post-operative fMRI session.

The MR imaging was performed with a 3.0 Signa HDx MR scanner at Haukeland University Hospital, Bergen. First, 3D volume images were acquired with a T1-weighted spoiled-gradient (SPGR) pulse sequence. Next, several series of fMRI BOLD data were acquired, using an echo planar imaging (EPI) sequence. The T1-weighted structural images were acquired in 188 sagittal slices, thickness = 1.0 mm, with a 256 × 256 matrix, echo time (TE) = 2.948 ms, repetition time (TR) = 7.736 ms, flip angle (FA) = 14°. The T1-weighted images were used for positioning the slices for subsequent definition of the functional EPI volumes parallel to the AC-PC with the following parameters: TE = 30 ms, TR = 3.0 s, FA = 90°, matrix = 128 × 128, ∼35 slices of thickness = 3 mm with 0.6 mm gap, using an interleaved acquisition procedure. Pixel size was 1.72 mm^2^, giving a 220 mm field-of-view (FOV). Five “dummy” scans were acquired at the beginning of each functional series in order to avoid confounding by initial arousal and other effects.

### Response Instructions and MRI Image Acquisitions

The participants went through a sequence of alternating OFF and ON blocks, following a classic box-car design, starting and ending with an OFF-block. Each block lasted for 30 s, which corresponded to 10 image volume acquisitions per block, and 90 volumes in total. The five OFF and four ON blocks were repeated for the four instruction runs, thus a session consisted of 9 × 30 s × 4 runs = 1080 s. The sequence of instructions for the four sessions was of the pattern: right elbow flexion movements, left elbow flexion movements, right key-grip movements, and left key-grip movements. For some of the patients, the actual pattern of instructed movements deviated somewhat from the specified sequence, depending on the specific nature of the surgical operation. Instructions were presented as auditory, verbal cues through a standard start-stop sequence implemented in nordicAktiva software (NordicNeuroLab, Inc., Bergen, Norway^1^), synchronized to block onset triggers.

### Pre-processing

The paradigm, by design and necessity, involves somewhat gross limb movements – this may inevitably lead to some additional head motion, and perhaps local magnetic field fluctuations associated with the changing limb position. Therefore, a processing pipeline which was well-suited to dealing with these movements and associated artifacts was required. For the present purposes a standard AFNI pipeline (generated by afni_proc.py) was utilized (Analysis of Functional NeuroImages, June 16, 2014 build, Robert W. Cox et al., NIMH Scientific and Statistical Computing Core; 1994–2014). EPI data were corrected for slice onset timing (using quintic interpolation of the time series data), then subject to motion-correction with strong outliers (Euclidean norm of the derivatives of motion parameters exceeding 0.3 mm per TR) censored. Slice-onset and motion-corrected functional data were then registered to the high-resolution structural image, aligned via this structural image to standardized space (Talairach and Tournoux, 1988), smoothed with a 4 mm FWHM kernel and intensity-normalized. Regression analysis was performed by a generalized additive model, given the block timing parameters and incorporating estimated motion parameters as additional regressors. This resulted in statistical maps in standard space, indicating the correspondence of each voxel with the stimulus condition, for each movement by each subject in both the pre- and post-operative state. These resultant F-maps formed the basis for all further processing; reports from the motion-correction and model estimation phases provided quantifiable metrics for data quality, to supplement visual inspection.

### Analysis

**FIGURE 1:**
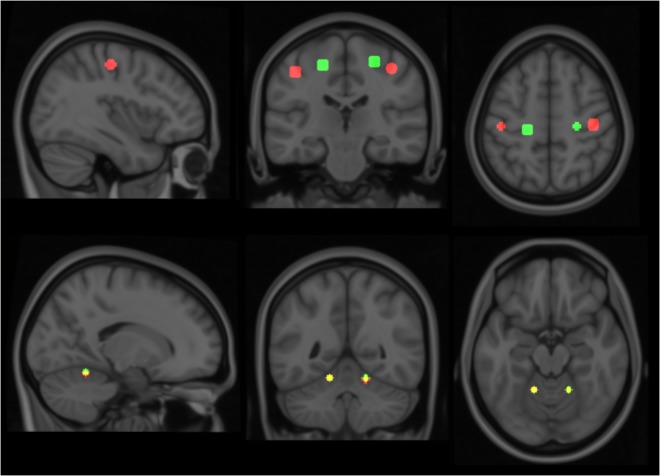
Modeled locations of primary activation. Regions associated with elbow flexion action are shown in green, those associated with key grip activity in Red. Colors are additive, i.e., yellow indicates overlap of elbow and key components. See text for further details.

Considering the small number and somewhat varied condition of the subjects, standard group-analyses for statistical significance would prove difficult. Therefore, a template model for each of the motor movements was derived based on data collected from three healthy controls; and individual patients were subsequently analyzed against this model. The healthy controls performed the same elbow and key grip movements while in the MR scanner as the patients were required to do. The resulting template model is shown in **Figure 1**, where expected spatial localization of activation to elbow movements are marked in green, and to key grip movements marked in red; regions where activations from elbow and key grip movements overlap are marked in yellow. The identified cerebral ROIs were then used as template regions for localization of activations to elbow and key grip movements, and used for calculations of percentage of overlap between actual and expected activations pre-and post-operatively in the patients.

### Model Definition

Taking data from the healthy, non-operated control subjects allowed typical spatial patterns of activation for each of the motor actions to be determined by means of cluster analysis. These activation maps were thresholded at *F* = 30 (roughly corresponding with *p*∼ = 0.06), for the null hypothesis of “voxel contains only noise,” as compared with the alternative hypothesis “voxel contains signal plus noise.”

For each map, the center-of-mass location of the five most significant clusters was noted, and any others discarded. Similar clusters across controls were then grouped according to proximity, using a locally implemented algorithm – each cluster was grouped with the nearest corresponding activation in other control subjects within a given radius threshold (10 mm), recursively until no further valid groupings were possible. This allowed for typical locations of strong activation common to all controls to be identified for each of the particular movement types, together with an estimation of inter-subject variation – as shown in **Table 1**.

**Table 1:** Typical center-of-mass for peak activations in healthy controls, for each action.

	Primary activation [mm in Talairach space (21)]					Secondary activation [mm in Talairach space (20)]				
	X	Y	Z	SD	Atlas location	X	Y	Z	SD	Atlas location
Left key grip	40.1	-21.8	50.6	3.5	Primary motor/pre motor	-16.8	-50.8	-16	2	Cerebellum, left V
Right key grip	-43.6	-23.7	48.4	3.8	Primary motor/somatosensory	15.95	51.65	-17.9	2.4	Cerebellum, right V
Left elbow flexion	25.23	-23.9	56.7	4	Primary motor/pre motor	-15.05	-51.6	-16	4.6	Cerebellum, left V
Right elbow flexion	-20.85	-25.95	54.3	3.3	Primary motor	15.2	-51.7	-16.15	1.3	Cerebellum, right V

*X, Y, and Z: stereotaxic coordinates according to Talairach (21): negative X-values = left side, Y-values = posterior, Z-values = inferior.*

For each of the actions, this method isolated two distinct regions of activation – one in the primary motor area and a second in the cerebellum. Relative locations of activations found within the primary motor area are consistent with long-established organizational maps (Penfield and Boldrey, 1937), and compatible with more recent MRI studies involving similar movements, for example (Meier et al., 2008). These locations show clear distinction between the elbow and key-grip actions (**Figure 1**); corresponding difference vectors are shown in **Table 2**.

**Table 2:** Typical difference-vectors across subjects, between left and right side for each movement action, and between elbow flexion and key-grip movement actions for each side.

Co-ordinates	X	Y	Z
Left-right side, key grip	83.7	2	2.2
Left-right side, elbow flexion	46.1	2.0	2.4
Elbow-key, left side	-14.8	-2.1	6.1
Elbow-key, right side	22.8	-2.2	5.9

*X, Y, Z co-ordinates refer to distances in mm, in Talairach space [21], see **Table 1**.*

Location of motor activity within the cerebellum is compatible with existing findings (Roland et al., 1980; Stoodley and Schmahmann, 2009), however, at this resolution and sample size no significant differences (apart from clear lateralization) could be identified between the different actions.

### Region-of-Interest (ROI) Analysis

Gaussian volumes-of-interest were defined to characterize each of the actions, centered around the locations identified in **Table 1** with sigma 7 mm. The choice of radius was guided by the standard deviation of location between subjects (<4 mm), and the observed separation between center-of-mass locations for the different actions (>16 mm); although not documented herein, our findings are nonetheless robust for a range of radii and a variety of kernel functions.

**FIGURE 2:**
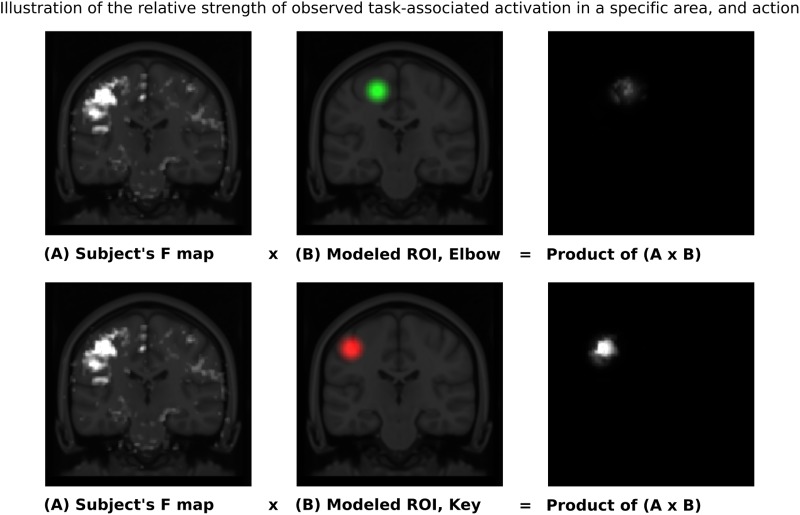
Sample ROI analysis for control person C3, Right Key grip action – showing clear dominance of activation in the region defined by the Key ROI. The images to the left show coronal slices through the motor cortex, with the actual observed activation to elbow flexion and key grip, respectively. The mid-images show the same coronal slices with the modeled areas for elbow (green) and finger (red) movements, respectively, based on anatomical data from the controls. The right-images show the product of the F-map (recorded activation) and the Gaussian volume-of-interest (modeled activation). The brighter the resulting product, the better the overlap between recorded and predicted activation for the respective area. See text for further explanations.

Given these characteristic regions, it was possible to compare the relative strength of observed task-associated activation in a specific area, for each action, by taking the product of the F-map and the Gaussian volume-of-interest. Thus, it was possible to classify each individual series using a simple binary comparison: is more task-associated activation measured within the modeled elbow region, or the modeled key-grip region? This process is illustrated in **Figure 2** for case C3. The different maps in **Figure 2** shows the F-map to the far left (A), the modeled ROI-map in the middle (B), and the resulting overlap map between A and B, by taking the product of the F-map and modeled ROI map, separate for the elbow (upper row) and key grip (lower row) movement actions. For case C3, which is illustrated in **Figure 2**, this resulted in 10.2% overlap for the elbow movement, and 38.2% for the key grip movement, thus, key grip was dominant post-operatively by 57.9%, i.e., [(38.2 - 10.2)/(38.2 + 10.2) ^∗^ 100] = 57.9. Using this approach we calculated the dominant localization for each subject and action pre- and post-operatively for the patients (P1–P4), and for the controls (C1–C3). The percentage of overlap between the F-map and ROI-map is shown in the “Key” and “Elbow” columns in **Table 3**, and the relative percentage of dominance of either action is shown in the far-right column “Rel%.”

**Table 3:** Outcome of region-of-interest based classification.

Subject	Side	Action	State	Key	Elbow	Expected	Dominant	Rel %
P1	Right	Elbow flexion (E)	Pre	15.0	21.3	E	E	17.2
P1	Right	Key grip (K)	Post	2.5	5.1	K	E	-33.6
P2	Right	Elbow flexion (E)	Pre	9.9	13.3	E	E	14.3
P2	Right	Key grip (K)	Post	6.4	3.6	K	K	27.3
P3	Left	Elbow flexion (E)	Pre	6.7	7.7	E	E	7.2
P3	Left	Key grip (K)	Post	17.4	10.6	K	K	24.4
P3	Right	Key grip (K)	Post	24.5	13.7	K	K	28.4
P3	Right	Elbow flexion (E)	Post	10.4	22.1	E	E	36.2
P3	Left	Elbow flexion (E)	Post	24.0	25.6	E	E	3.1
P4	Right	Key grip (K)	Pre	60.7	21.7	K	K	47.3
P4	Right	Key grip (K)	Post	32.5	12.0	K	K	46.1
C3	Right	Elbow flexion (E)	Control	6.5	14.9	E	E	39.0
C3	Left	Elbow flexion (E)	Control	8.3	20.6	E	E	42.9
C3	Right	Key grip (K)	Control	38.2	10.2	K	K	57.9
C3	Left	Key grip (K)	Control	38.3	31.2	K	K	10.3
C1	Right	Elbow flexion (E)	Control	3.3	4.0	E	E	8.7
C1	Left	Elbow flexion (E)	Control	9.6	23.6	E	E	42.1
C1	Right	Key grip (K)	Control	31.4	7.5	K	K	61.4
C1	Left	Key grip (K)	Control	97.8	45.8	K	K	36.2
C2	Right	Elbow flexion (E)	Control	28.1	31.8	E	E	6.1
C2	Left	Elbow flexion (E)	Control	26.5	41.7	E	E	22.4
C2	Right	Key grip (K)	Control	7.6	2.6	K	K	48.9
C2	Left	Key grip (K)	Control	34.3	10.9	K	K	51.8

*P = patient, C = healthy control, E = Elbow movement, K = key-grip movement, Pre = preoperative, Post = post-operative. Section “Materials and Methods” for further explanation of the column headings.*

## Results

**FIGURE 3:**
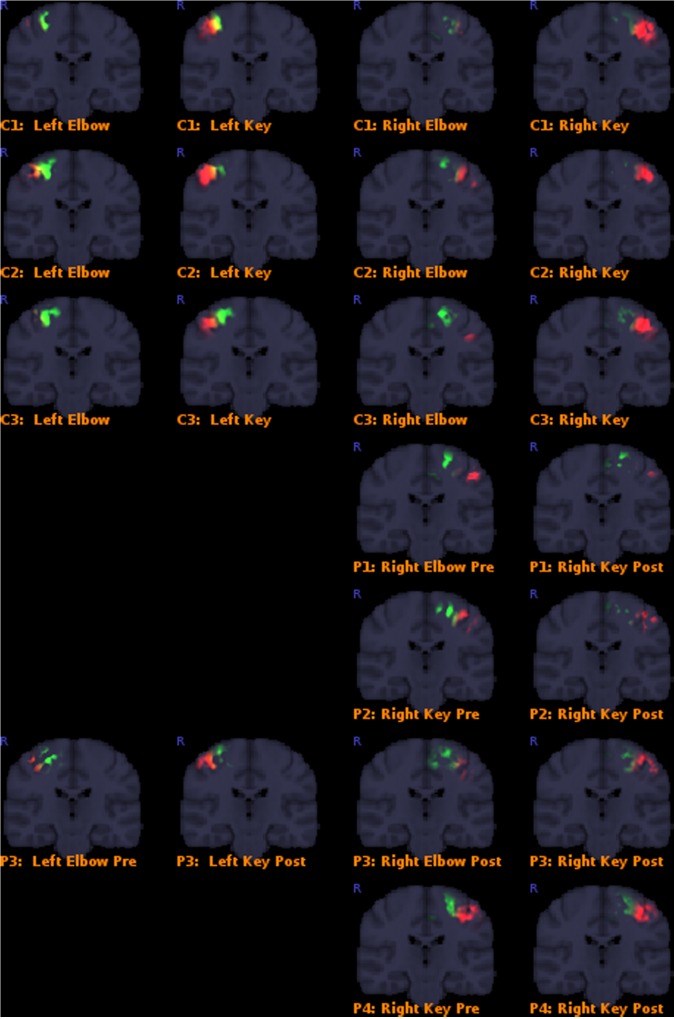
Colored areas show the actual masked BOLD contrast data for each participant. P = patients, C = controls. Activation intersecting with the Elbow models are shown in green, while activations intersecting with the Key grip model are shown in red.

Results overall indicated that the post-operative key-grip movement in the tetraplegic patients was elicited from a similar brain region as in healthy controls, despite an alternative muscle (musculus brachioradialis, normally associated only with elbow movements) being deployed. Details of the results are presented in **Table 3**, and corresponding **Figure 3**. This supports our hypothesis that control of that muscle shifts from a brain region typically associated with elbow movement, to one typically associated with wrist movements.

Quality reports from the pre-processing phase confirmed acceptable data quality across all series, with few if any time points in any given acquisition rejected due to excessive motion. There were two exceptions: the pre-operative elbow flexion data from subjects P2 and P3 had a quite high proportion (∼30%) of frames censored due to excessive motion. Nonetheless the statistical outcomes were sufficiently robust as to remain usable despite the omission of affected frames, and the results (per below) appear unperturbed.

Classification outcomes from the region-of-interest analysis are presented in **Table 3**. For all control subjects, the classification matched expectations, i.e., the dominant region identified by the analysis, corresponded with the model for the expected action being performed, as illustrated in **Figure 2** for case C3. Furthermore, post-operative outcomes for all pre-operative acquisitions from patients P1–P4 matched expectations, and were according to the hypothesis. This serves to confirm the validity of the derived model and the region-of-interest based classification approach. See further details in **Table 3**.

Post-operative data from patient P1 defied expectations, implying that the modeled elbow region remained dominant post-operatively in eliciting the key-grip movement. However, this acquisition fit relatively poorly to the model in the first place, and furthermore cluster-based functional analysis identified no large, significant regions of activity in this case. Hence, the significance of this contrary result is doubtful.

Post-operative data from patient P2 was consistent with expectations: key-grip motion post-operatively was found to be elicited predominantly from a brain region matching the modeled key-grip region.

Similarly for P3, who had earlier been operated on the right side to provide a key-grip function, and now had undergone a similar operation on the left side: movement of fingers on both the previously-operated right side and the newly-operated left side, corresponded with activation dominant in the modeled key-grip area.

In the case of P4, for whom a pre-existing key grip was reinforced, results are inconclusive. In both the pre- and post-operative cases, activation was most dominant in the modeled key-grip region as expected, and presented a similar distribution between cases; activation in the post-operative case was weaker, however.

## Discussion

As mentioned in the Introduction, it has been shown that cortical sensory-motor areas appear to be relocated after long-term sensory and motor deprivation in chronic patients, such as amputees. Forced training in humans with acquired or congenital neurological deficits, such as stroke or unilateral cerebral palsy, have also been shown to alter cortical organization, however only with enlargement of sensory-motor areas, but not a spatial relocation (Inguaggiato et al., 2013; Gauthier et al., 2014).

To our knowledge, the findings presented here are the first indications that the human cortex is capable of reorganizing itself spatially as a consequence of surgically altered motor periphery after transposition of a muscle tendon from the arm to the hand. This post-operative cortical reorganization, with the representation of the elbow flexor moving “down” from the preoperative elbow area toward the cortical hand area, appears to reflect the process behind the effect of the training to use the muscle in a new way, to obtain a hand grip instead of flexion of the elbow. Moreover, this cortical reorganization seems to occur within the time frame of the patients’ “learning” of the new movement, i.e., months after the tendon transposition.

As pointed out above, the present findings only give an indication that such a relocation of cortical representation may take place. Thus, the main limitation of the present study is the restricted number of enrolled patients. Before any definite conclusions can be drawn on this matter, the present findings must be reproduced in a larger sample. A potential limitation of the findings is that the binary comparison of activations associated with either elbow or key-grip movements could overlook that there was mutual activations occurring. We cannot rule out this possibility, and one would not expect *all* activation to shift to the key-grip area after surgery, but rather that the overall gravity of activation would shift, which the results also show.

## Ethics Statement

All procedures performed in studies involving human participants were in accordance with the ethical standards of the institutional and/or national research committee and with the 1964 Helsinki declaration and its later amendments or comparable ethical standards.

The study was approved by the Regional Committee for Medical Research Ethics in Western Norway (REK Vest) (#2012/2220). The consent obtained by the participants was both informed and written.

## Author Contributions

KH contributed in planning, running, analysis, and writing up the study. KW contributed in planning, patient recruitment, running, and writing up of the study. LH contributed in planning, patient recruitment, running, and writing up of the study. AC contributed in planning, running, analysis, and writing up of the study. RB contributed in planning, running, and analyzing the study.

## Conflict of Interest Statement

KW, AC, and RB owns shares in the company NordicNeuroLab, Inc. which produced add-on equipment for the fMRI recordings. The other authors declare that the research was conducted in the absence of any commercial or financial relationships that could be construed as a potential conflict of interest.

## Footnotes

1 http://www.nordicneurolab.com/
